# Unique Case Report of a Meningeal Sarcoma Arising during Ongoing Treatment for Progressing Intraparenchymal Glioma

**DOI:** 10.1155/2019/7950782

**Published:** 2019-11-27

**Authors:** Richard A. Peterson, Bhavani Kashyap, Pamala A. Pawloski, Anna C. Forsberg, Leah R. Hanson

**Affiliations:** ^1^Regions Hospital Cancer Care Center, St Paul, MN, USA; ^2^HealthPartners Neuroscience Center, St Paul, MN, USA; ^3^HealthPartners Institute, Minneapolis, MN, USA

## Abstract

Radiation-induced sarcomas in the brain are extremely rare, usually occur with an average latency of 9 years, and are associated with poor outcomes. Latency periods shorter than 1 year may indicate a genetic predisposition such as Li-Fraumeni syndrome. A 34-year-old man underwent initial tumor resection and radiation therapy for a World Health Organization (WHO) Grade II Astrocytoma. Within 6 months, the tumor recurred as WHO Grade III and was treated with temozolomide and then bevacizumab. Despite the patient's apparent improving condition, MRI revealed new dural-based lesions 10 months after radiation therapy and identified as high-grade sarcoma. The patient resumed bevacizumab, began NovoTTF treatment for progressing glioma, and ifosfamide/doxorubicin for the sarcoma. Genetic testing revealed no pathogenic mutation in the TP53 gene. Ultimately, treatment was unsuccessful and the patient succumbed to glioma and sarcoma within 2 years of initial diagnosis. This case was unique due to the rapidly progressing glioma and sudden appearance of a high-grade sarcoma. It is unusual to have two separate intracranial primary cancers with each requiring a different chemotherapy regimen. We discuss the difficulty of simultaneously treating with separate chemotherapy regimens. It remains unclear whether the sarcoma was induced by the radiation treatment or a genetic predisposition.

## 1. Introduction

Ionizing radiation is a common treatment for a variety of intracranial tumors. However, radiation therapy carries the known risk of inducing secondary malignancies [1–3]. CNS sarcomas arising independently following radiation therapy for an intraparenchymal primary tumor are extremely rare and usually occur after a latency of many years [4]. Shorter latency periods may indicate a genetic predisposition such as Li-Fraumeni syndrome. Unfortunately, radiation-induced sarcomas generally result in poor outcomes and low survival rates [5], highlighting the need for effective treatment. In this report, we describe a unique case of an intracranial sarcoma that developed less than one year after radiation therapy for a primary glioma and during simultaneous chemotherapy treatment for the progressing primary tumor.

## 2. Case Presentation

**Figure 1:**
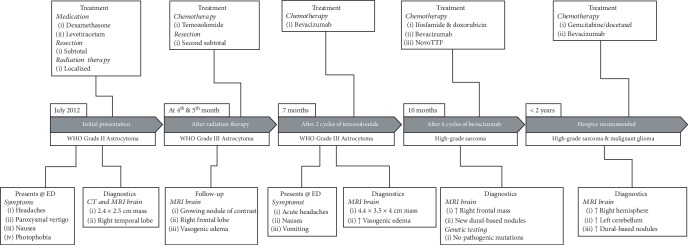
Timeline of the case report.

**Figure 2:**
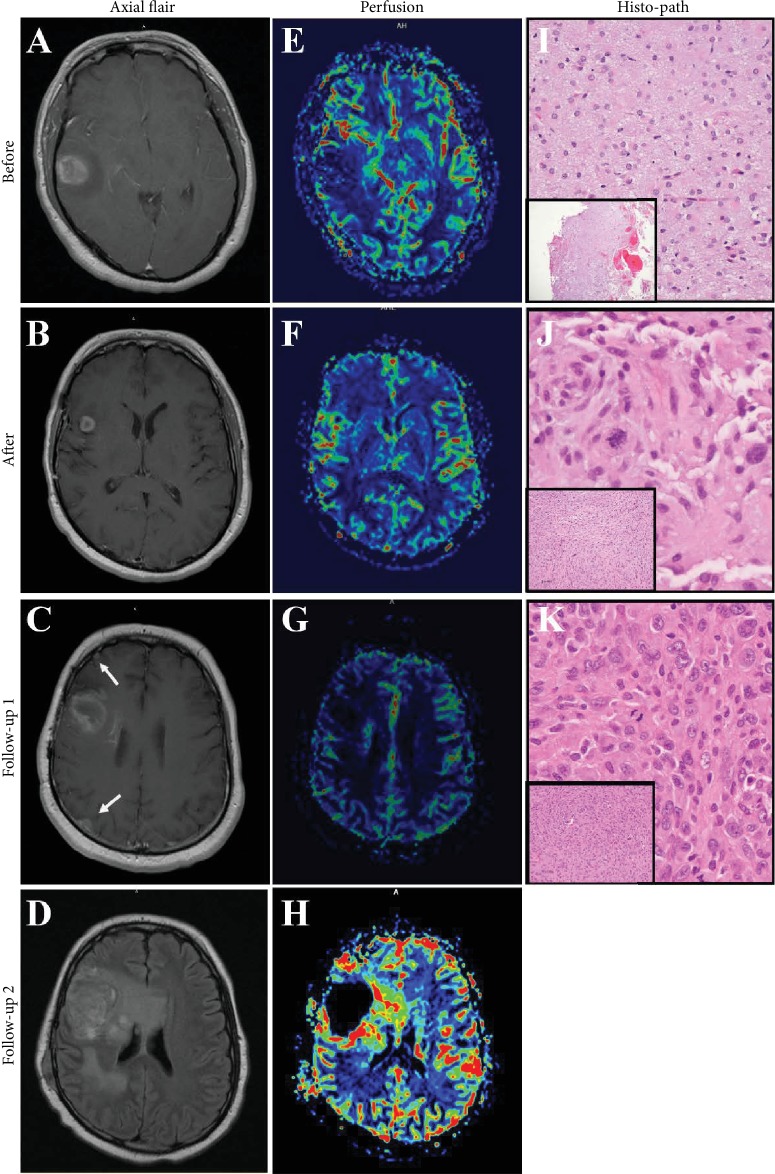
The progression of astrocytoma and development of dural-based nodules. (a–d) Images of axial MRI: a 2.4 × 2.5 cm mass in the right lateral to midtemporal lobe and associated area of hemorrhage at initial presentation (a), a growing nodule of contrast enhancement in the right frontal lobe at 5 months after completion of RT (b), follow-up 1 after 6 cycles of bevacizumab (c), and an increase in dural-based nodules involving the entire right hemisphere and the left cerebellum as well as an increase in size of the centrally necrotic mass in the right frontal lobe at follow-up 2 of dural-based nodules (d). White arrows indicate new dural-based nodular areas of enhancement. (e–h) Images of MRI perfusion: at initial presentation (e), at 5 months after completion of RT (f), at follow-up 1 after 6 cycles of bevacizumab (g), and at follow-up 2 of dural-based nodules (h). (i–k) Representative images of H&E staining: an initial Grade II Astrocytoma at high and low (inset) power (i), dural nodule showing increased mitotic activity at high and low (inset) power (k), and Grade III Astrocytoma at high and low (inset) power after second tumor resection (j).

In July 2012, a 34-year-old man presented to the Emergency Department with a 4-month history of headaches, paroxysmal vertigo, nausea, and photophobia, with acute worsening of symptoms over the previous week. His past medical history was unremarkable, and there was no significant family history or any occupational exposure potentially associated with sarcoma.  Figure 1 provides a timeline of events including diagnostic assessments and interventions. Computed tomography (CT) and magnetic resonance imaging (MRI) of the brain demonstrated a 2.4 × 2.5 cm mass in the right lateral to midtemporal lobe and associated area of hemorrhage (Figures 2(a) and 2(e)). Dexamethasone was prescribed for cerebral edema and levetiracetam for seizure prophylaxis. A subtotal resection was achieved using a right temporal craniotomy and stealth-guided tumor resection. Pathologic review identified a WHO Grade II Astrocytoma (Figure 2(i)). The patient was treated with localized radiation therapy (RT) with 5040 cGy which was well-tolerated. No chemotherapy regimen was started at this time.

Repeated MRI at 4 and 5 months after completion of RT showed a growing nodule of contrast enhancement in the right frontal lobe and associated vasogenic edema despite dexamethasone treatment (Figure 2(b)). MRI perfusion studies showed increased blood flow to these new areas (Figure 2(f)), and a second tumor resection was performed. Pathologic assessment revealed a mixture of radiation necrosis and WHO Grade III Astrocytoma (Figure 2(j)) with no isocitrate dehydrogenase (IDH) mutation. The patient began a regimen of temozolomide (cycle 1 : 150 mg/m^2^, days 1-5 each 28-day cycle; cycle 2 : 175 mg/m^2^). After two cycles of temozolomide treatment, the patient presented to the Emergency Department with a 1-week history of acute headache, nausea, and vomiting. MRI revealed a 4.4 × 3.5 × 4 cm heterogeneously enhancing mass lesion with perfusion, consistent with progressing tumor. Imaging also showed a significant increase in surrounding vasogenic edema which proved difficult to manage due to increasing complications from dexamethasone treatment, including persistent folliculitis and myopathy.

**Figure 3:**
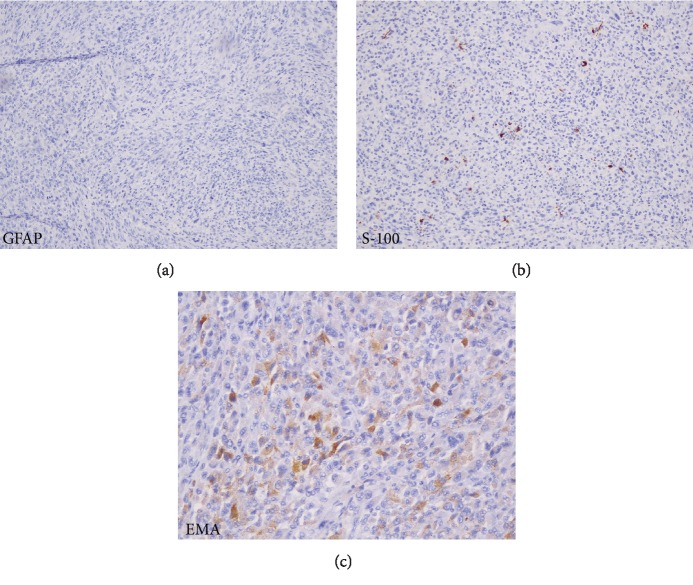
Dural-based high-grade sarcoma without differentiating features. (a) Representative image of GFAP stain showing no features of a glial neoplasm. (b) Representative image of a positive S100 staining indicative of a stromal tumor. (c) Representative image of EMA stain, suggestive of epithelial characteristics of the tumor.

The patient's treatment regimen was changed to bevacizumab (10 mg/kg every 2 weeks) to treat the underlying tumor and vasogenic edema, in an effort to reduce corticosteroid exposure. The patient tolerated 6 cycles of bevacizumab well, and his clinical condition began to improve. However, a follow-up MRI showed increased enhancement of the right frontal lobe mass and new dural-based nodular areas of enhancement (Figure 2(c)). Clinically, the patient appeared to be responding to bevacizumab. As expected, there was reduced cerebral blood flow observed on MRI perfusion; however, the increased enhancement was concerning as this is typically reduced with bevacizumab treatment (Figure 2(g)). An initial concern was there may be dissemination of high-grade glioma. A lumbar puncture was deferred due to mass effect. A biopsy of a right frontal dural-based nodule was pursued, and the pathologic assessment was consistent with a high-grade sarcoma lacking any further differentiating features (Figures 2(k) and 3(a)–3(c)). While the type of sarcoma could not be determined, it was specifically *not* gliosarcoma. After presentation of the sarcoma, the patient was referred to a genetic counselor with concern for a genetic predisposition (specifically, Li-Fraumeni syndrome). However, genetic testing revealed no pathogenic mutations in the TP53 gene or any other known mutations.

Further imaging of the neuroaxis was completed to ensure there was no spread of the sarcoma through the subarachnoid space. MRI of the cervical, thoracic, and lumbar spine showed no areas of abnormal enhancement, and a CT PET of the body revealed no other new hypermetabolic lesions. Multidisciplinary discussions with medical and radiation oncologists ensued regarding the number and spread of the dural-based lesions, and chemotherapy was considered to be the best treatment option for the sarcoma. The patient was started on a regimen consisting of ifosfamide (3750 mg/m^2^ days 1-2 each 21-day cycle) and doxorubicin (30 mg/m^2^ days 1-2 each 21-day cycle), continuation of bevacizumab (10 mg/kg every 2 weeks; 4 additional cycles), and initiation of NovoTTF (Optune) treatment for the malignant glioma. The chemotherapy treatment was not well-tolerated, and the treatment course was complicated by neutropenic fever and fatigue despite growth factor support and dose reduction. Thus, the patient only completed 3 cycles. Data from the Optune device showed sporadic use, averaging less than 18 hours per day.

Ultimately, the patient's condition began to further deteriorate. Follow-up MRI revealed an increase in dural-based nodules involving the entire right hemisphere and left cerebellum, as well as an increase in size of the centrally necrotic mass in the right frontal lobe (Figures 2(d) and 2(h)). The recommendation was made to initiate hospice care. The patient received a second opinion at another institution, where he received a gemcitabine/docetaxel regimen, but only completed one dose of gemcitabine due to thrombocytopenia and clinical worsening. The patient received one additional dose of bevacizumab (15 mg/kg). He continued to decline and eventually succumbed to glioma and sarcoma less than 2 years after his initial diagnosis.

## 3. Discussion

We presented a unique case of a patient who underwent tumor resection and radiation therapy for a WHO Grade II Astrocytoma. This case was unusual due to the rapidly progressing glioma and sudden appearance of a high-grade sarcoma. This presented the unique challenge of simultaneously treating two distinct intracranial malignancies requiring different chemotherapy regimens. Upon progression of Grade II Astrocytoma following resection and radiation therapy, consensus guidelines recommended chemotherapy, reirradiation, or palliative care [6].

The clinical progression of gliomas is typically intraparenchymal; leptomeningeal spread is less common. The incidence of symptomatic leptomeningeal or extracranial metastasis from glioma is rare, estimated at 2% based on case series reports [7–13]. When such spread of glioma does occur, patients typically deteriorate rapidly [12]. However, at the appearance of dural-based lesions, this patient's clinical appearance had been improving with bevacizumab treatment, indicating that the dural-based nodules may have originated from a separate process. This was confirmed by biopsy, which revealed a high-grade sarcoma.

Primary central nervous system sarcomas are rare. Case reports document a variety of histological subtypes, including malignant fibrous histiocytoma, chondrosarcoma, rhabdomyosarcoma, liposarcoma, fibrosarcoma, angiosarcoma, leiomyosarcoma, and epithelioid sarcoma [14–26], and the incidence of each is extremely low. In this case, pathologic review from two separate institutions could not determine a particular subtype for the sarcoma; however, it was specifically not gliosarcoma.

The appearance of the sarcoma could have indicated an effect of the radiation treatment; however, the risk of developing a radiation-induced tumor after fractionated radiation therapy in the CNS is estimated at only 1-3% within 30 years [27–29]. Meningiomas and gliomas are the most common reported radiation-induced malignancies [30–32]. Sarcomas occur in about 0.03%-0.3% of patients treated with radiation and make up about 12% of radiation-induced cancers of the head and neck [33–35], usually manifesting as malignant fibrous histiocytomas or fibrosarcomas [36]. Our literature search for postradiation intracranial sarcomas identified 22 additional cases with a latency that ranged from 13 months to 19 years with a mean latency of 8.6 years. Of those, 17 (73.9%) experienced latencies of 6 years or longer [2–4, 35, 37–43]. The range of radiation dosages reported was 3600-6300 cGy, with most sarcomas occurring after treatment with 5100-6000 cGy (11/23, 47.8%). This patient received 5040 cGy.

While there is no standard criteria for defining a sarcoma as radiation-induced, our case does not fit the popular description by Cahan et al. [44], which specified that the sarcoma begin more than one year after radiation. One similar case, reported by Alotaibi and Petrecca, of a 47-year-old man treated with whole brain radiation therapy for a glioblastoma, had a latency of 13 months before the induction of the sarcoma [45]. This case was a single tumor that was resected surgically and subsequently treated with radiotherapy followed by doxorubicin chemotherapy and a positive outcome. CNS sarcomas arising independently after radiation therapy for an intraparenchymal primary tumor are extremely rare and usually result in poor outcomes. Reported 5-year survival rates are estimated at less than 30% [34]. Our literature review [2–4, 35, 37–43, 45, 46] confirmed that, whether treated by radiation, surgery, chemotherapy, or a combination, the 10-year survival rate was 17.4% (4/23 patients).

Alternatively, the presence of two separate cancerous processes could suggest a genetic predisposition. Given the patient's young age at diagnosis and the combination of astrocytoma and sarcoma, there was concern for the possibility of Li-Fraumeni syndrome. Li-Fraumeni is a rare hereditary disorder linked to mutations of the tumor protein p53 (TP53) gene and increases the risk of developing multiple cancers early in life (commonly, breast cancers, sarcomas, and brain tumors) [47]. However, genetic testing revealed no pathogenic mutations in the TP53 gene. While this does not completely rule out the possibility of a genetic predisposition, the lack of known family history of cancers makes it less likely.

It is worth noting that the patient's initial treatment was administered before the results of the RTOG 9802 trial were released. These results showed the addition of PCV (procarbazine, lomustine, and vincristine) chemotherapy after adjuvant RT improved overall survival for low-grade glioma [48]. Further treatment recommendations for disease recurrence include bevacizumab and chemotherapy (temozolomide, nitrosoureas, carboplatin, cyclophosphamide, etoposide, or irinotecan). The patient's tumor was negative for IDH-1, which is found to be mutated in 43% of diffuse low-grade astrocytomas [49] and is associated with better outcomes [50]. Of additional interest is the *MGMT* promoter and 1p/19q status, which was not available for this patient because coverage for the tests was denied by the patient's insurance carrier. Deleted 1p/19q and/or *MGMT* hypermethylation in anaplastic gliomas imparts better prognosis for patients treated with radiotherapy or chemotherapy [51–53].

Although the patient received standard therapy for sarcomas (ifosfamide and doxorubicin), in an era of targeted agents, one would consider treatment directed at both tumor types. There are data suggesting both tumor types could be treated with the same agent. For example, an EGFR mutation that occurs in roughly 40% of glioblastomas and also known to be highly expressed in many sarcomas would be an ideal target. Unfortunately, the EGFR inhibitors have been shown to be largely ineffective [54]. Similarly, other targeted agents that are used for the treatment of sarcoma include imatinib [55, 56], pazopanib [57, 58], sorafenib [59, 60], and sunitinib [61–63] and also have shown minimal effect in glioma.

CNS penetration with ifosfamide and doxorubicin is variable, and these agents are not effective for the treatment of glioma [64], thereby making this case therapeutically complex. The influence of the two disease processes, glioma and sarcoma, within the CNS could potentially impact the integrity of the BBB; however, the degree of which is unknown and limited because of existing interstitial fluid gradients [65]. It is also possible that the bevacizumab therapy administered to the patient for the treatment of his recurrent glioma may have enhanced delivery of the doxorubicin by lowering the interstitial fluid pressure [66].

Overall, this case illustrates the difficulty of simultaneously treating two separate intracranial malignant cancers: one intraparenchymal and the other dural-based. Each tumor required a different chemotherapy regimen. After thorough review, providing any treatment required simultaneous therapy for these two separate malignant processes.
